# B cells and the humoral response in melanoma: The overlooked players of the tumor microenvironment

**DOI:** 10.1080/2162402X.2017.1294296

**Published:** 2017-03-03

**Authors:** Giulia Chiaruttini, Silvia Mele, James Opzoomer, Silvia Crescioli, Kristina M. Ilieva, Katie E. Lacy, Sophia N. Karagiannis

**Affiliations:** a St. John's Institute of Dermatology, Division of Genetics and Molecular Medicine, Faculty of Life Sciences and Medicine, King's College London, Guy's Hospital, London, UK; b NIHR Biomedical Research Centre at Guy's and St. Thomas's Hospitals and King's College London, King's College London, London, UK; c Breast Cancer Now Research Unit, Division of Cancer Studies, Faculty of Life Sciences and Medicine, King's College London, Guy's Hospital, London, UK

**Keywords:** Antibodies, B cells, humoral response, immunoglobulins, immunosurveillance, melanoma, tumor microenvironment

## Abstract

Evidence of tumor-resident mature B cell and antibody compartments and reports of associations with favorable prognosis in malignant melanoma suggest that humoral immunity could participate in antitumor defense. Likely striving to confer immunological protection while being subjected to tumor-promoting immune tolerance, B cells may engender multiple functions, including antigen processing and presentation, cytokine-mediated signaling, antibody class switching, expression and secretion. We review key evidence in support of multifaceted immunological mechanisms by which B cells may counter or contribute to malignant melanoma, and we discuss their potential translational implications. Dissecting the contributions of tumor-associated humoral responses can inform future treatment avenues.

## Malignant melanoma and immune responses in the clinical landscape

Rising incidence (global incidence reported in 2013 in 15–25 individuals in every 100,000) and the worst patient survival rates of all skin tumors continue to make malignant melanoma treatment clinically challenging, despite recent breakthroughs in targeted therapies.^1^ Multiple moles, family history of melanoma and unprotected or intense exposure to UV light,^2^ especially UVB-induced somatic DNA mutations, such as cytidine to thymidine (C>T) transitions,^3^ are among the risk factors. Mutation of genes such as BRAF and NRAS involved in the MAPK kinase pathway are present in more than 50% of the melanoma tumors.^4-6^ Until 5 y ago, development of distant metastases was generally associated with a historic median survival of less than one year.^7^ Recent breakthroughs in the understanding of the molecular and immunological basis of melanoma have contributed to the development of new MAPK pathway inhibitors, small molecule inhibitor drugs and checkpoint blockade antibody treatments, improving clinical outcomes in subsets of patients.^8^

Reports of tumor-resident and systemic immune responses in melanoma patients, clinical observations of partial lesion regressions and spontaneous remissions, increased rates of malignant melanoma in immunosuppressed patients (organ transplant recipients and HIV-infected individuals),^9^^,^^10^ as well as partial successes of early immunostimulating treatments such as interleukin-2 (IL-2) and interferon-α2b (IFNα-2b) reported over many years, together support the presence of an active immune surveillance in patients with melanoma.^11^^,^^12^ Investigations into the drivers of immune responses to melanoma elucidated not only a set of tumor-specific melanoma-antigen-encoding gene families (MAGE, BAGE, GAGE), but also several antigenic epitopes derived from human melanocyte lineage-specific proteins (MART-l/Melan-A, gpl00, gp75 and tyrosinase) recognized by CD8^+^ and CD4^+^ T cells.^13-15^ Various peptide-based vaccination therapy approaches have been trialed in patients using these antigens, often in conjunction with cytokines, toll-like receptor (TLR) agonists and adjuvants, some demonstrating circumscribed success.^16^ Evidence for a correlation between antitumor T cell responses^17-19^ and heightened lymphocytic infiltrates within melanoma lesions^20^ with longer patient survival have maintained interest in the search for therapies based on counteracting peripheral tolerance. Several personalized therapeutic approaches have been developed for melanoma involving adoptive cell therapy (ACT) with T cells.^21-23^ Some promising outcomes have been reported in small-scale studies of patients with malignant melanoma treated with autologous tumor-infiltrating lymphocyte (TIL)-based ACT, with larger, randomized phase III clinical trials to ascertain broader clinical benefits still required.

More recently, immunotherapeutic antibodies that block immune checkpoint molecules have led to the regulatory approval of the anti-cytotoxic T lymphocyte antigen-4 (CTLA-4) antibody ipilimumab for the treatment of metastatic melanoma,^24^^,^^25^ followed by the anti-programmed cell death protein 1 (PD1) receptor antibodies nivolumab and pembrolizumab.^26^^,^^27^ These agents function through blocking inhibitory molecules on the T cell surface, thereby counteracting immune suppressive signals.^28^^,^^29^ In 2016, the FDA approved the drug atezolizumab for bladder and lung cancers. Atezolizumab is an inhibitor of the PD1 ligand (PD-L1), expressed on tumor cells that is thought to restrict T cell activation through recognition and engagement with PD1 on T cells.^30^ This has opened the way for several phase I clinical trials currently looking at efficacy of this class of agents in patients with melanoma. The emergence of these checkpoint inhibitor antibodies has been an important clinical breakthrough, bringing cancer immunotherapy to the forefront of clinical oncology. Numerous reports have drawn correlations between patient responses to checkpoint inhibitors and the presence and nature of tumor-infiltrating T lymphocytes and T cell responses to melanoma tumor antigens.^31^

Although insights into the roles of T cells in antitumor responses have been widely studied and accepted as an essential immunological dimension in anti-cancer immunity, the roles of B cells and of the humoral response remain insufficiently elucidated. B cells confer a broad array of functions, which include antigen processing and presentation, cytokine-mediated signaling, immune regulation, expression and secretion of antibodies. These attributes can contribute to antitumor immunity and to treatment responses or, on the other hand, tumors can co-opt inhibitory immune pathways aimed at maintaining B cell immune tolerance. The characteristics affecting these opposite outcomes still need to be fully uncovered. Here, we review evidence from human clinical investigations and from murine cancer models in support of multifaceted immunological mechanisms by which B cells may respond or contribute to melanoma.

## B cells in melanoma tumor inflammation

### B cell infiltration

Lymphocyte populations are found in and around many solid tumor lesions including melanomas. While T cells are the most prominent, infiltrating human B cells are increasingly being reported in melanomas and other tumor types.^32-37^

In an immunohistochemical study of 106 primary human melanoma samples, the majority of tumor tissues contained significant amounts of infiltrating CD20^+^ cells, thought to be B lymphocytes, dispersed in the stroma immediately around the tumor.^37^ Denser B cell infiltration correlated with activated T cells, which could imply potential activation of an antitumor response. The percentage of both intra-tumoral and peri-tumoral B cell infiltration also positively correlated with patient survival, since patients' samples with significantly higher levels of B cell infiltration failed to develop visceral metastasis over the subsequent 5-y window of observation.^37^ In concordance, a study following a patient cohort over 5-y since primary melanoma tumor resection, found that initial larger B cell infiltration in primary tumors of patients positively correlated with subsequent improved disease-free survival after cancer vaccine therapy.^38^ A similar correlation was found in a study on two cohorts of 57 and 41 patients with primary cutaneous melanoma.^39^ An earlier report, however, found no significant correlation between B cell infiltration and survival, although there was a significant association between total infiltrating TILs and disease progression in a cohort of 58 patients with malignant melanoma.^40^ A study from a cohort of 91 patients with a clinical follow up of at least 10 y found instead an increased tumor progression and decreased overall survival in those patients with more than 15% B cell density among TILs in primary cutaneous melanoma.^41^ Possible explanations for these discrepancies among studies could include the different tumor locations analyzed (primary cutaneous melanomas, distal or lymph node metastases), the way data were analyzed (cell frequencies reported as absolute numbers or as a proportion of TILs) and B cell detection markers (e.g., the pan-B cell marker CD20 alone may not be sufficient). The latter aspect may be crucial toward building meaningful correlations and a wider understanding of the B cell subsets and their markers would probably be required.

Although these observations support the idea that the level of B cell infiltration may, overall, be representative of the host's potential to develop antitumor responses, it remains to be determined whether higher lymphocyte infiltration is a result of a tumor antigen-specific immune response or merely recruitment of lymphocytes to tumor microenvironments in response to inflammation. Such contrasting findings point to a complex relationship between humoral responses and tumors.

### B cells in human melanoma-associated tertiary lymphoid structures (TLS)

**Figure 1.**
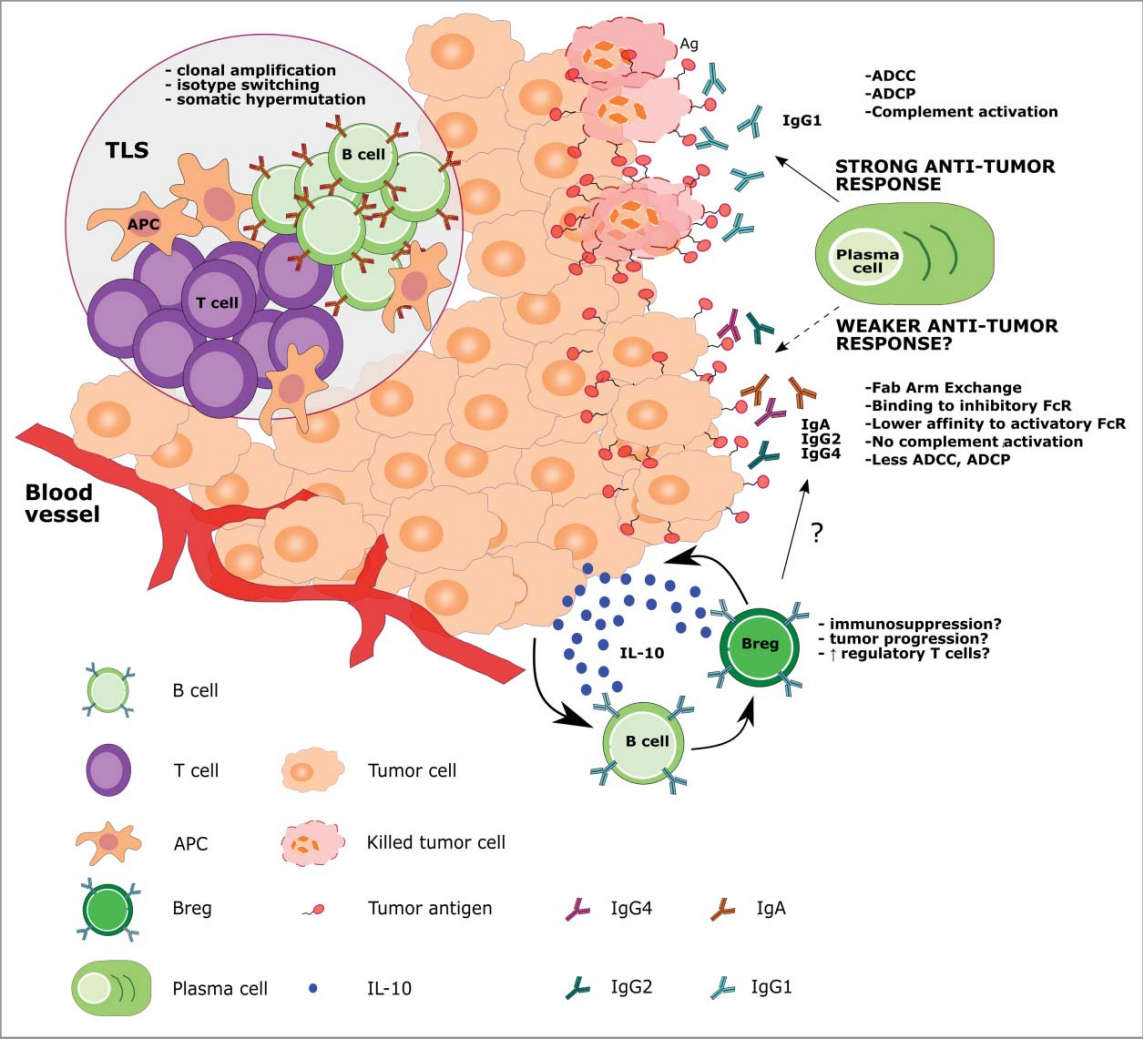
Proposed B cell functions in the melanoma tumor microenvironment. B cells may arise from the local immune surveillance environment or migrate to the tumor from blood vessels. B cells may accumulate and expand in tumor-associated lymphoid structures (TLS), where they can encounter APCs and T cells, and undergo affinity maturation and clonal amplification. Within the tumor, plasma cells can secrete tumor-specific IgG1 antibodies, effective in inducing ADCC, ADCP and complement-mediated cytotoxicity. On the other hand, in the tumor microenvironment, B cells can be differentially activated to secrete antibody isotypes such as IgA, IgG2 and IgG4, which may induce a weaker immune response through (a) inability to activate the complement cascade, (b) lower affinities for activatory FcRs, (c) higher affinities for inhibitory FcRs, (d) lower potency in triggering ADCC and ADCP compared with IgG1 isotype antibodies and (e) in the case of IgG4, Fab-arm exchange, resulting in antibodies with low antigenic affinity. The tumor microenvironment may also differentially polarize B cells toward a regulatory phenotype (Breg) through the secretion of soluble factors such as IL-10, which, in turn, negatively influences immune cell activation and antibody class switching.

Activation of lymphocytes during adaptive immune responses typically occurs in secondary lymphoid structures such as lymph nodes, spleen and mucosal-associated lymphoid tissues. Chronic inflammation, however, can be accompanied by the formation of tertiary lymphoid structures (TLS) at lesion sites in both mouse models and humans.^42^ These structures can vary in size and organizational structure, from disordered mixtures of dendritic cells (DCs), T and B cells to highly ordered structures bearing striking resemblance to germinal centers typically found in lymphoid organs.^43^ TLS can be found at sites of inflammation in almost any organ of the body and are thought to facilitate rapid and robust immune responses^44^ (Fig. 1).

As tumor lesions bear characteristics consistent with chronic inflammation, the presence of tumor-associated TLS, which contain B cell infiltrates, reported in different tumor types including melanomas, may not be surprising.^37^^,^^45^ In a study of 106 primary human melanoma lesions, 26% contained histologically visible aggregates.^37^ Cipponi et al. recovered and micro-dissected highly ordered TLS, defined as lymphoid follicles which contained clusters of B cells, follicular DCs, T cells and mature DCs from 7 out of 29 human melanoma metastases.^45^ Sequencing of the immunoglobulin (Ig) repertoire of the lymphoid follicles revealed clonal amplification, isotype switching and somatic hypermutation, suggesting a local antigen-driven response^45^ (Fig. 1). These hallmarks of local B cell maturation have also been observed in immunohistochemical analyses of extra-nodal TLS from human germ cell tumors^46^ and breast carcinomas.^47^ In an analysis of TLS in non-small cell lung cancer, characterization of B cell subsets by immunohistochemistry and flow cytometry detected a prevalence of memory B cells and plasma cells producing tumor-specific antibodies, with the density of the follicle correlating with the number of mature plasma cells present.^48^ In concordance, TLS were found to be prognostic of more favorable patient outcomes.^43^^,^^48^

Collectively, these findings may indicate a role of TLS as local sites of B cell maturation, fostering the generation of *in situ* adaptive host immune responses.

### Evidence for B cells and their functions in experimental models

#### Murine models of melanoma and other tumors

Initial studies in murine models of melanoma and of other cancers suggest that B cells may exert both pro- and antitumor effects, often depending on the *in vivo* model system. Reported tumor-permissive properties of B cells include B cell-dependent inhibition of antitumor immunity in lymphoma and melanoma (but not in sarcoma), through a CD40L-dependent mechanism that affects IL-10 secretion *in vitro*.^49^ Other studies provide evidence that B cells may support lymphangiogenesis in *in vivo* lymphoma and melanoma mouse models^50^ and angiogenesis *in vitro* and also *in vivo* in melanoma, bladder and lung carcinoma murine tumor models.^51^ In a murine model of squamous cell carcinoma, antitumor autoantibodies were reported to induce acute inflammation when organized in immune complexes. According to this study, the inflammatory environment regulates recruitment and induces pro-tumoral functions of leukocytes surrounding neoplastic tissue through engagement of Fc gamma receptors (FcγRs) expressed by immune cells^52^ (Fig. 1). These pro-tumoral functions engendered by an abnormal secretion of Ig could be reversed by administration of an anti-CD20 treatment in a combined therapy with a chemotherapy agent, which ablated B cells, reprogrammed the chemokine expression profiles of macrophages and increased CD8^+^ T cell infiltration into mouse tumors.^53^ In contrast, several other studies suggest that B cells can augment T cell-mediated antitumor responses in *in vivo* models of melanoma, lymphoma, colorectal and mammary carcinoma.^54-58^

These *in vivo* studies not only suggest that B cells can strongly contribute to tumor rejection, but also acquire tolerant or pro-tumorigenic characteristics with disease progression (Fig. 1). It is therefore tempting to envisage a complex orchestration of the immune response mediated by different B cell subsets, perhaps including B cells with immunoregulatory properties, as is the case for different T cell subsets.

#### The search for regulatory B cells (Bregs): insights from animal models

**Figure 2.**
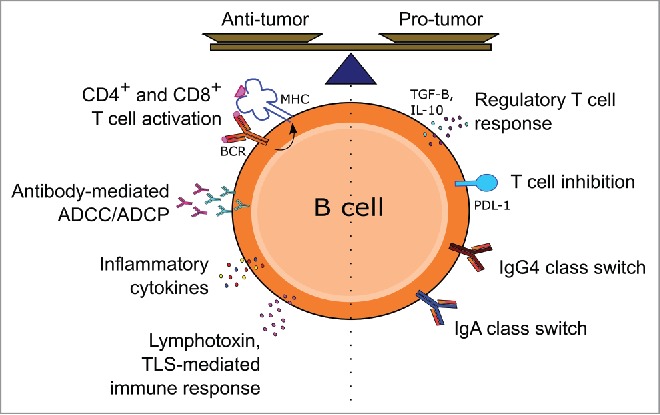
Potential pro- and antitumor functions of tumor-infiltrating B cells. Tumor-infiltrating B cells may either promote or inhibit growth and metastasis through various immune mechanisms, involving secretion of antibodies, cytokine-mediated activation and recruitment of other immune effector cells and engagement and activation of T cells through antigen presentation via MHC in the presence of co-stimulatory molecules. Regulatory functions may be engendered through secretion of cytokines such as IL-10, T cell inhibition by PD-L1 expression or class switching and production of immunoglobulin isotypes with low immune effector stimulating functions.

Mizoguchi et al. first described a subset of gut-associated CD1d-expressing B cells that could suppress inflammatory progression of colitis in mice by secreting the immune regulatory cytokine IL-10, thus coining the term “regulatory B cell” (B10)^59^ (Figs. 1 and 2). In later studies, B10-like IL-10-producing B cells were reported in peripheral human blood^60^ and early findings suggest that these cells may also be present in human metastatic melanoma.^61^ However, possible roles of regulatory B10-like B cells in cancer have to-date only been described in animal models.^62^^,^^63^ A study in a transgenic murine model of prostate cancer identified PD-L1 and IL-10, expressed by a subpopulation of plasma cells, as the factors responsible for CTL inhibition after treatment with the immunogenic chemotherapeutic drug oxaliplatin.^64^ Bregs have also been shown to regulate immunity to murine breast tumors independently of IL-10 *in vivo* and *in vitro*.^65^^,^^66^ Although the exact mechanism of this IL-10-independent immune suppression is yet unknown, some evidence links Breg activity and CD25^+^ FoxP3^+^ T regulatory cell accumulation at tumor sites in murine breast tumors.^66^^,^^67^ Moreover, a subset of murine B220^+^ CD19^+^ CD25^+^ Bregs was shown to be enriched in 4T1 breast tumor-bearing mice and to polarize T cells in culture toward a regulatory T cell (Treg)-like phenotype, again in an IL-10-independent manner.^65^ Tumor-derived Bregs and Tregs were consequently suggested to interact with myeloid-derived suppressor cells, possibly further potentiating Treg responses, thus amplifying immunosuppression and promoting disease progression.^65^ In the study of Khan et al., Bregs expressing high levels of PD-L1 were observed to inhibit development and expansion of follicular T helper cells in an autoimmune disease *in vivo* model in mice and *ex vivo* in human blood, resulting in reduced B cell maturation and T cell-dependent humoral immune responses^68^ (Fig. 2).

Although pointing to potential roles for Bregs in tumor immune escape, results obtained in animal models are yet to be fully confirmed and elucidated in the human melanoma patient context.

## B cells in melanoma immune surveillance

### Evidence for reactive mature B cell responses and tumor-specific antibodies

B cells straddle both innate and adaptive immunity, acting as critical effectors of the humoral immune response through the secretion of antibodies.^69^ In several cancer types, TILs and peripheral B cells have the ability to produce antibodies that could recognize autologous tumor targets, some of which have been investigated as potential diagnostic biomarkers.^70-72^ The development of the serological identification of recombinant expression cloning (SEREX) approach, a phage display of cDNA libraries derived from tumor samples screened with autologous cancer patient sera, constituted a powerful tool that allowed the identification of more than one hundred melanoma antigens and autoantibodies to these. Findings from SEREX studies supported the notion that tumors such as melanoma are immunogenic and induce temporal tumor-reactive humoral responses.^73^^,^^74^ However, whether tumor-reactive antibodies *in vivo* have any antitumor protective functions remains under debate. Mature B cells from melanoma patients were able to produce IgG antibodies that recognize melanoma cells, and *ex vivo* these antibodies could mediate tumor cell cytotoxicity^75^ (Fig. 2). There is also evidence of a gradual reduction of the human B cell compartment and of tumor-reactive antibodies with melanoma disease progression. This may indicate some functional roles for B cells early on in the disease which may perhaps be modulated as a part of tumor-associated immune escape processes.^76^ In this regard, Oaks and colleagues described for several human cancers an active anti-inflammatory role of sialylated tumor-specific IgGs that may promote tumor growth.^77^

Saul et al. report higher mRNA expression of mature B cell markers in human melanoma lesions compared with normal skin and a distinct affinity-matured antibody repertoire. Antibodies from melanoma lesions featured shorter complementarity-determining region 3 (CDR3) sequences, clonal expansion characteristics and differential antigen recognition patterns (demonstrated by homology modeling), suggesting a distinct melanoma-associated B cell response^78^ (Fig. 1).

Emerging evidence suggests that the state of maturation of the B cell compartment and subsequent isotype expression of tumor-reactive antibodies can prevent the host from mounting an efficient immune response.^79^ Depending on the soluble signals released in the tumor microenvironment, B cells can be polarized to undergo class switching and express potentially weak immune-activating antibody isotypes such as IgG4^80^ or IgG2 and IgA,^45^ as part of chronic inflammation and immune-escape processes associated with human melanoma^79^ (Figs. 1 and 2). It is noteworthy that a subtype of murine B cells (B-1 cells) have been shown to secrete IgM antibodies in a T cell-independent manner after recognition of evolutionally conserved structures of microbial origin through TLR *in vitro*.^81^^,^^82^ T cell-independent and BAFF-dependent activation has been reported to support production of IgG4 antibodies by human B cells in autoimmune pancreatitis.^83^ Such innate antibody response triggers may also be envisaged in the context of melanoma tumors, and may give rise to B cell expansion in the absence of specific tumor antigen signal.

IgG4, one of the antibody isotypes produced in melanomas, is known to have poor immune-activating properties, including inability to activate complement,^84^ a lower capacity to mediate effector functions compared with other isotypes^85^ and phagocytosis (ADCP) compared with IgG1. These have been associated with specific structural characteristics of IgG4, which determine its poor capacity to bind C1q, its poor affinity for activating receptor FcγRIIa (known to be involved in antibody-dependent macrophage-mediated phagocytosis, ADCP),^86^ and FcγRIIIa (crucial for NK cell-mediated ADCC functions).^87^ Structural characteristics of IgG4 are also responsible for relatively high affinity to the inhibitory Fc receptor FcγRIIb, compared with other subclasses, and ability to undergo Fab-arm exchange with other IgG4 molecules.^88-90^ Collectively, through such functional attributes, IgG4 isotype antibodies are thought to have immunomodulatory properties, and to impair antibody effector functions in cancer.^80^^,^^91^ Elevated IgG4 levels in patient circulation even in the early stages of melanoma has been associated with disease progression and less favorable clinical outcome.^80^^,^^92^ IgG4^+^ B cells and IgG4s were detected in melanoma tumor microenvironments together with IL-10, IL-4 and vascular endothelial growth factor (VEGF). *Ex vivo* co-culture experiments with human B cells and melanoma cells suggest that melanoma cells have the ability to promote Th2-biased conditions to support IgG4 production by B cells.^80^^,^^92^ (Figs. 1 and 2).

Human melanoma lesion-resident B cells have also been reported to express IgA class antibodies.^45^ IgA class switching is normally observed in secondary lymphoid organs that drain mucosal tissue and is associated with a tolerance induction to commensal microbiota in the gut.^93^ IgA class switching is often associated with chronic exposure to antigens and this could facilitate a form of immune subversion mediated by the inflammatory milieu, much like that of the IgG4 class switching. IgA is a poor inducer of complement, ADCC and ADCP.^93^ A study in a murine model of prostate cancer describes a subset of plasma cells expressing IgA in the tumor region and having immune regulatory functions.^64^ Yet, more work is needed to investigate the role human IgA response plays in tumor immunity (Figs. 1 and 2).

Based on our current knowledge, it is possible that, depending on immune context and microenvironment, B cells will be able to respond to cytokine stimuli in the presence or absence of specific antigenic challenge, to either promote or counteract tumor development. The tumor microenvironment could signal B cells in germinal centers and TLSs to undergo skewed class switching, leading to the secretion of Th2-biased isotypes and thus dampening immune response.

### Could B cells act as antigen-presenting cells to enhance T cell responses?

As professional antigen presenting cells (APC), B cells are able to induce antigen-specific T cell priming, which requires both recognition of antigens by their Ig-membrane bound B cell receptor (BCR), as well as engagement of the co-stimulatory molecule CD40 by a CD4^+^ helper T cell. This is followed by B cell maturation, antigen internalization, processing and presentation on major histocompatibility complexes (MHC)^94-98^ (Fig. 2).

Depending on their activation status, B cells can also secrete an array of cytokines, notably tumor necrosis factor-α (TNF-α), IL-10, lymphotoxin (LT), IL-2, IL-6, IL-4 and interferon-γ (IFNγ). Through cytokine secretion, B cells can exert dynamic effects on both the local microenvironment and the systemic immune response^99-103^ (Fig. 2). In allograft rejection, potent T cell responses are a pivotal component in pathogenesis and tissue destruction. Allograft tissue-reactive B cells can enhance T cell response through antigen presentation and co-stimulation.^104,105^ Gene-expression profile studies in renal allograft biopsies, corroborated by immunohistochemical analyses, have shown that B cell signatures (comprising of CD20, CD74 and Ig) are associated with acute organ rejection. Similarly, immunohistochemical evaluations revealed dense interstitial CD20^+^ B cell aggregates in 53% (17/32) of core biopsy samples with graft rejection.^105^

B cell activation may also be critical for tumor regression in melanoma. Primary human B cells activated *in vitro* with CD40 ligand and subsequently pulsed with melanoma tumor antigens, efficiently processed and presented MHC class II-restricted peptides to specific CD4^+^ T cell clones, generating melanoma-specific T cells.^106^ *Ex vivo* studies by Von Bergwelt-Baildon et al. on human blood-derived lymphocytes also suggest that in the context of tumor immunology, B cells have the ability to operate as efficient APCs, driving the expansion of both memory and naive tumor-associated antigen-specific CD4^+^ and CD8^+^ T cells^96^ (Fig. 2). B cells have also been reported to possess direct cytotoxic killing ability against murine 4TI breast cancer cells in a Fas/FasL-dependent manner in the absence of IL-10^107^ and also in the presence of IL-2.^108^

B cells may thus potentially contribute a wide variety of functions, including antigen presentation, to promote autoimmunity or tumor rejection.

## Therapeutic avenues focused on B cells

Although our understanding of the crosstalk between humoral immunity and melanoma remains incomplete, several therapeutic treatments have been attempted with a view of modulating the B cell compartment to stimulate anticancer immunity. The anti-CD20 monoclonal antibody (mAb) rituximab was administered in 15 patients with renal cell carcinoma and 6 with melanoma before treatment with low doses of IL-2 in a clinical trial, without conferring any beneficial effects.^109^ In the context of other tumor types, a case study of a primary cutaneous T cell lymphoma showed a temporary remission after a combination therapy with rituximab and chemotherapy, associated with decrease in Tregs and increase in CD8^+^ T cells.^110^ In a pilot study on a small cohort of nine stage IV melanoma patients, treatment with rituximab increased the median time without recurrences from 6 to 42 mo (no recurrence in 5 out of 9 patients at 42 mo).^111^ No correlation was found though between patients with recurrences and immune cell responses. The authors suggested that treatment benefits are due to the antibody's ability to target a subpopulation of CD20-expressing cancer stem cells instead. There are currently several on-going clinical trials testing antibodies targeting CD20 for the indication of metastatic melanoma (e.g., NCT01376713, NCT02142335; www.clinicaltrials.gov). It is the authors' opinion that, apart from anti-CD20 treatments potentially eliminating cancer stem cell populations, generally targeting a widely expressed molecule such as CD20 does not take into account the complex contributions of different B cell subpopulations in cancer. Thus, more fine-tuned approaches to perhaps target specific subpopulations of B cells (e.g., regulatory or IL-10-producing B cells) or inhibitory elements on B cells could constitute possible strategies.

CD40–CD40L interactions have also been the focus of some immunotherapies aimed at driving B cell proliferation and producing sufficient quantities of B cells *in vitro* for adoptive therapy.^112^ CD40L-stimulated CD40-expressing B cells can be expanded from small volumes of blood and have been reported to generate antitumor CD8^+^ T cells.^113^ In a preclinical setting, adoptive transfer of B cells isolated from draining lymph nodes of 4TI tumor-bearing mice and activated *in vitro* with LPS and anti-CD40, have been shown to prevent spontaneous metastasis of 4TI breast tumor cells to mouse lungs.^114^ Given encouraging results from clinical trials and regulatory approval of the adoptive T cell therapy sipeleucel T for the treatment of prostate cancer, it is tempting to consider the possibility that, in future, activated B cells could be used as an adjuvant in adoptive T cell therapies.

## Insights from the humoral response in melanoma for the development of future immuno-oncology treatments

B cells play multifaceted roles in melanoma immunity through several signaling and immunological pathways. In this review, we showed numerous evidences supporting this concept, also making parallels with different disease models. Yet, further studies will be needed to dissect the mechanisms by which different components of B cell functions operate in melanoma, and thus offering the potential to identify previously unappreciated translational insights. Initial approaches may involve in-depth dissection of specific B cell subsets and modulatory markers which may affect cell differentiation, migration, functions, antibody expression, maturation, class switching and secretion. Immunologically relevant disease models and *ex vivo* systems could in future also help delineate specific mechanisms which could be responsible for immunosuppression or for activation of humoral immunity in melanoma.

As melanoma cells may be able to escape host immune responses,^115-117^ the B cell repertoire and antibodies expressed in the patient context may not be effective enough to confer tumor clearance. Antibody isotypes such as IgG4 and IgA may regulate immune effector functions and support immune evasion. Melanoma-associated IgG4 could perhaps act by restricting Fc-mediated functions of IgG therapeutic antibodies in the circulation and in tumor lesions. These mechanisms should be taken into consideration when designing antibody therapeutic agents. For example, recently approved mAbs for the treatment of melanoma target immune checkpoint molecules on T cells and are designed to act through their Fab-mediated effects, by removing T cell inhibitory signals. However, the potency and mechanisms by which mAbs may also engage immune effector cells through their Fc regions are less well understood. Given the array of Fc receptors expressed by effector cells, including those infiltrating tumors, it may be important to understand how antibodies and effector cells that form part of tumor immune surveillance may influence antitumor immunity, and how they affect the efficacy of therapeutic antibodies.^118^ Furthermore, it is also tempting to consider new therapeutic opportunities through the design of therapeutic antibodies perhaps less prone to cancer-associated immunosuppressive forces or those better equipped to mount effector functions in the Th2-biased tumor microenvironment. These may include engineered antibodies with enhanced binding to activatory Fc receptors on immune effector cells to redirect them against cancer, or those antibodies with Fc regions of different isotypes such as IgE, perhaps better able to exert immunological surveillance in tissue tumors such as melanoma.^119^

Underpinning effective therapeutic interventions in melanoma will be the ability to avoid potential tumor blockade mechanisms and enable the host to mount a robust immune response toward a heterogeneous tumor population. In light of encouraging advancements with the use of individual and combination treatments with checkpoint blocking agents, the potential for using B cells and the antibodies they express for therapy or as prognostic or predictive biomarkers of treatment responses remain largely unexplored possibilities. In future, delineating the presence of different subsets of B cells and their impact on immune responses against melanoma could provide opportunities, such as targeting specific populations for elimination (e.g., Bregs or IL-10-producing B cells) or activation (e.g., tumor antigen-specific mature memory B cells), or the use of particular B cell subsets in adoptive therapy regimens. For instance, studies describing checkpoint molecules such as PD-L1 expressed by certain subsets of B cells raise the possibility to exploit specific checkpoint inhibitors to target modulated cell subsets as a strategy that could potentially re-kindle the antitumor functions of these cells. Furthermore, designing molecules that counteract immunosuppressive cytokines such as IL-10, VEGF or TGF-ß from the tumor microenvironment could revert a wider group of immune cells, including B cells, to engage in tumor rejection. Removal of these cytokines may allow B cells to undergo class switching to activatory antibody isotypes (e.g., IgG1), perhaps better able to engage effector cells against tumors. A wider immunotherapy or vaccination approach may also aim to re-establish the antitumor functions of different immune cell populations, to achieve simultaneous stimulation of tumor-neutralizing CTL and humoral immune responses against cancer antigens.

With melanoma continuing to provide the paradigm for clinical translation of cancer treatments based on triggering the activatory potential of T cells, more in-depth focusing on other immune cells including B cells and harnessing their antitumor functions may be of key importance for the development of the next generation of immuno-oncology agents. Dissecting B cells and the patients' humoral immunity and understanding how different compartments may contribute to tumor inflammation, immune responses and clinical course, warrant renewed attention and offer the possibility to widen the scope of immune-based therapies for melanoma and other cancers.
